# A Systematic Review of *In vitro* and *In vivo* Activities of Anti*-Toxoplasma* Drugs and Compounds (2006–2016)

**DOI:** 10.3389/fmicb.2017.00025

**Published:** 2017-01-20

**Authors:** Mahbobeh Montazeri, Mehdi Sharif, Shahabeddin Sarvi, Saeed Mehrzadi, Ehsan Ahmadpour, Ahmad Daryani

**Affiliations:** ^1^Toxoplasmosis Research Center, Mazandaran University of Medical SciencesSari, Iran; ^2^Student Research Committee, Mazandaran University of Medical SciencesSari, Iran; ^3^Department of Parasitology and Mycology, Sari Medical School, Mazandaran University of Medical SciencesSari, Iran; ^4^Department of Pharmacology, School of Medicine, Iran University of Medical Sciences TehranIran; ^5^Drug Applied Research Center, Tabriz University of Medical SciencesTabriz, Iran

**Keywords:** *Toxoplasma gondii*, toxoplasmosis, drugs, compounds, *in vitro*, *in vivo*

## Abstract

The currently available anti-*Toxoplasma* agents have serious limitations. This systematic review was performed to evaluate drugs and new compounds used for the treatment of toxoplasmosis. Data was systematically collected from published papers on the efficacy of drugs/compounds used against *Toxoplasma gondii* (*T. gondii*) globally during 2006–2016. The searched databases were PubMed, Google Scholar, Science Direct, ISI Web of Science, EBSCO, and Scopus. One hundred and eighteen papers were eligible for inclusion in this systematic review, which were both *in vitro* and *in vivo* studies. Within this review, 80 clinically available drugs and a large number of new compounds with more than 39 mechanisms of action were evaluated. Interestingly, many of the drugs/compounds evaluated against *T. gondii* act on the apicoplast. Therefore, the apicoplast represents as a potential drug target for new chemotherapy. Based on the current findings, 49 drugs/compounds demonstrated *in vitro* half-maximal inhibitory concentration (IC_50_) values of below 1 μM, but most of them were not evaluated further for *in vivo* effectiveness. However, the derivatives of the ciprofloxacin, endochin-like quinolones and 1-[4-(4-nitrophenoxy) phenyl] propane-1-one (NPPP) were significantly active against *T. gondii* tachyzoites both *in vitro* and *in vivo*. Thus, these compounds are promising candidates for future studies. Also, compound 32 (*T. gondii* calcium-dependent protein kinase 1 inhibitor), endochin-like quinolones, miltefosine, rolipram abolish, and guanabenz can be repurposed into an effective anti-parasitic with a unique ability to reduce brain tissue cysts (88.7, 88, 78, 74, and 69%, respectively). Additionally, no promising drugs are available for congenital toxoplasmosis. In conclusion, as current chemotherapy against toxoplasmosis is still not satisfactory, development of well-tolerated and safe specific immunoprophylaxis in relaxing the need of dependence on chemotherapeutics is a highly valuable goal for global disease control. However, with the increasing number of high-risk individuals, and absence of a proper vaccine, continued efforts are necessary for the development of novel treatment options against *T. gondii*. Some of the novel compounds reviewed here may represent good starting points for the discovery of effective new drugs. In further, bioinformatic and *in silico* studies are needed in order to identify new potential toxoplasmicidal drugs.

## Introduction

*Toxoplasma gondii (T. gondii)*, an obligate intracellular, parasitic protozoan, is the etiologic agent of toxoplasmosis. About 30–50% of the world population is infected with the parasite, and it is the most prevalent infection among humans (Tenter et al., 2000; Flegr et al., 2014). Worldwide, over 1 billion people are estimated to be infected with *T. gondii* (Hoffmann et al., 2012). Its prevalence in some countries is high (e.g., Brazil, 77.5%; Sao Tome and Principe, 75.2%; Iran, 63.9%; Colombia, 63.5%; and Cuba, 61.8%) (Pappas et al., 2009). The annual incidence of congenital toxoplasmosis was estimated to be 190,100 cases globally (Torgerson and Mastroiacovo, 2013).

In the United States, the Centers for Disease Control and Prevention (CDC) reported that 22.5% of the population 12 years and older have been infected with *Toxoplasma* with 1.1 million new infections each year, making it the second most common cause of deaths due to foodborne diseases (an estimated 327 deaths) and the fourth leading cause of hospitalizations attributable to foodborne illness (an estimated 4428 hospitalizations). Also, an estimated 400–4000 infants are born with congenital toxoplasmosis in the United States each year (Jones et al., 2014).

*T. gondii* has three infectious stages of sporozoites (in oocysts), tachyzoites (rapidly multiplying form), and bradyzoites (tissue cyst form). Among them, tachyzoites are responsible for clinical manifestations and the acute phase of the disease. They are susceptible to the immune response of the host and to drug action. The resistant cyst form of the parasite is resistant to both the immune system and drugs (Hill and Dubey, 2002).

Acute toxoplasmosis in healthy individuals is usually subclinical and asymptomatic, but may lead to chronic infection. However, toxoplasmosis can lead to great morbidity and mortality rates in imunocompromised or congenitally infected individuals (Dubey and Jones, 2008; Ahmadpour et al., 2014). In AIDS patients, presence of the parasite causes necrotizing encephalitis and focal cerebral lesions in the central nervous system (CNS) from primary or recrudescent infection. In immunocompetent patients, latent toxoplasmosis occurs with the formation of cysts principally in the CNS (Martins-Duarte et al., 2006).

In the recent years, the development of well-tolerated and safe specific immunoprophylaxis against toxoplasmosis is a highly valuable goal for global disease control (Lim and Othman, 2014). Immunotherapeutics strategies for improving toxoplasmosis control could either be a vaccine which would induce strong protective immunity against toxoplasmosis, or passive immunization in cases of disease recrudescence. In the last few years, much progress has been made in vaccine research on DNA vaccination, protein vaccination, live attenuated vaccinations, and heterologous vaccination; while there were few candidates on passive immunization. New vaccine candidates have been tested, including in particular proteins from *T. gondii* ROP, MIC, and GRA organelles, multi-antigen vaccines, novel adjuvants but until now the researches could not access to a proper vaccine for prevention of toxoplasmosis in human (Zhang et al., 2013, 2015).

The recommended drugs for treatment or prophylaxis of toxoplasmosis are pyrimethamine and sulfadiazine. Unfortunately, these drugs have side effects such as neutropenia, severe drop of platelet count, thrombocytopenia, leucopenia, elevation in serum creatinine and serum liver enzymes, hematological abnormalities, and hypersensitivity reactions (Bosch-Driessen et al., 2002; Silveira et al., 2002; Schmidt et al., 2006). In addition, other drugs, such as azithromycin, clarithromycin, spiramycin, atovaquone, dapsone, and cotrimoxazole (trimethoprim-sulfamethoxazole), have been used for clinical toxoplasmosis. However, these drugs are poorly tolerated and have no effect on the bradyzoite form (Araujo and Remington, 1992; Petersen and Schmidt, 2003; Serranti et al., 2011).

In a clinical trial, 24% of sera positive women treated with spiramycin and pyrimethamine plus sulfadoxine combination delivered *Toxoplasma* infected infants in France (Bessières et al., 2009). Spiramycin monotherapy can be effective during the early stage of pregnancy to prevent prenatal transmission (Julliac et al., 2010). More than 50% of patients treated with spiramycin retained *T. gondii* DNA in blood and remained infected (Habib, 2008).

In recent years, studies have focused on finding safe drugs with novel mechanisms of action against *T. gondii*. Accordingly, there is an urgent need to evaluate new drugs based on novel and innovative therapeutic strategies against *T. gondii* that are both efficacious and nontoxic for humans (Rodriguez and Szajnman, 2012; Vanagas et al., 2012; Angel et al., 2013). Therefore, the goal of the present systematic review was to retrieve published studies related to *in vitro* and *in vivo* evaluation of drugs and compounds for the treatment of toxoplasmosis (2006–2016) in order to prepare comprehensive data for designing more accurate investigations in future.

## Methodology

This review followed the preferred reporting items for systematic reviews (PRISMA) guidelines (Moher et al., 2009).

### Literature search, study selection, and data extraction

English databases, including PubMed, Science Direct, Scopus, Google Scholar, ISI Web of Science, and EBSCO, were systematically searched for papers on *in vitro* and *in vivo* evaluation of anti-*Toxoplasma* activities of drugs and compounds, published worldwide, from 2006 to 2016. The keywords included were: “Toxoplasmosis,” “*T. gondii*,” “Anti-*Toxoplasma*,” “Drug,” “Anticoccidial,” “Treatment,” “*In vitro*,” “*In vivo*,” and “Compound.”

Papers written in English were selected. Gray literature and abstracts of articles which were published in congresses were not explored. In addition, in order to avoid missing any articles, whole references of the papers were meticulously hand-searched. Among English articles found with the mentioned strategies, full text papers that used laboratory method both *in vitro* and *in vivo* were included.

Also, studies with at least one of the following criteria were excluded: (1) studies that were not relevant; (2) articles not available in English; (3) studies on treatments for ocular infection; (4) articles that were of review or descriptive study type; (5) articles which contained no eligible data; (6) case series reports; (7) the data were duplicated from other studies or we were unable to obtain them; (8) those that were on efficacy of anti-*T. gondii* medicines in humans; and (9) any drug with an IC_50_ value > 10 μM.

### Data collection

All the experimental studies that were carried out to evaluate the efficacy of either drugs or compounds against *T. gondii* both *in vitro* and *in vivo* were included, and replicates were excluded. The inclusion criteria for selection of *in vitro* studies were important information about medication used for the experiments, type of cells used for culture, identification of the *Toxoplasma* strain, laboratory methods used for assessing drug activities, and main results comprising of the 50% inhibitory concentration (IC_50_). We reported *in vivo* studies used animal models, *Toxoplasma* strain, route of infection, the treatment schedule (dosage, route of administration, duration of treatment), the criteria for assessing drug activity (mainly survival for acute toxoplasmosis, histology, and brain cyst burdens for chronic infection), and the main results.

## Results

### Analysis of the included literature

**Figure 1:**
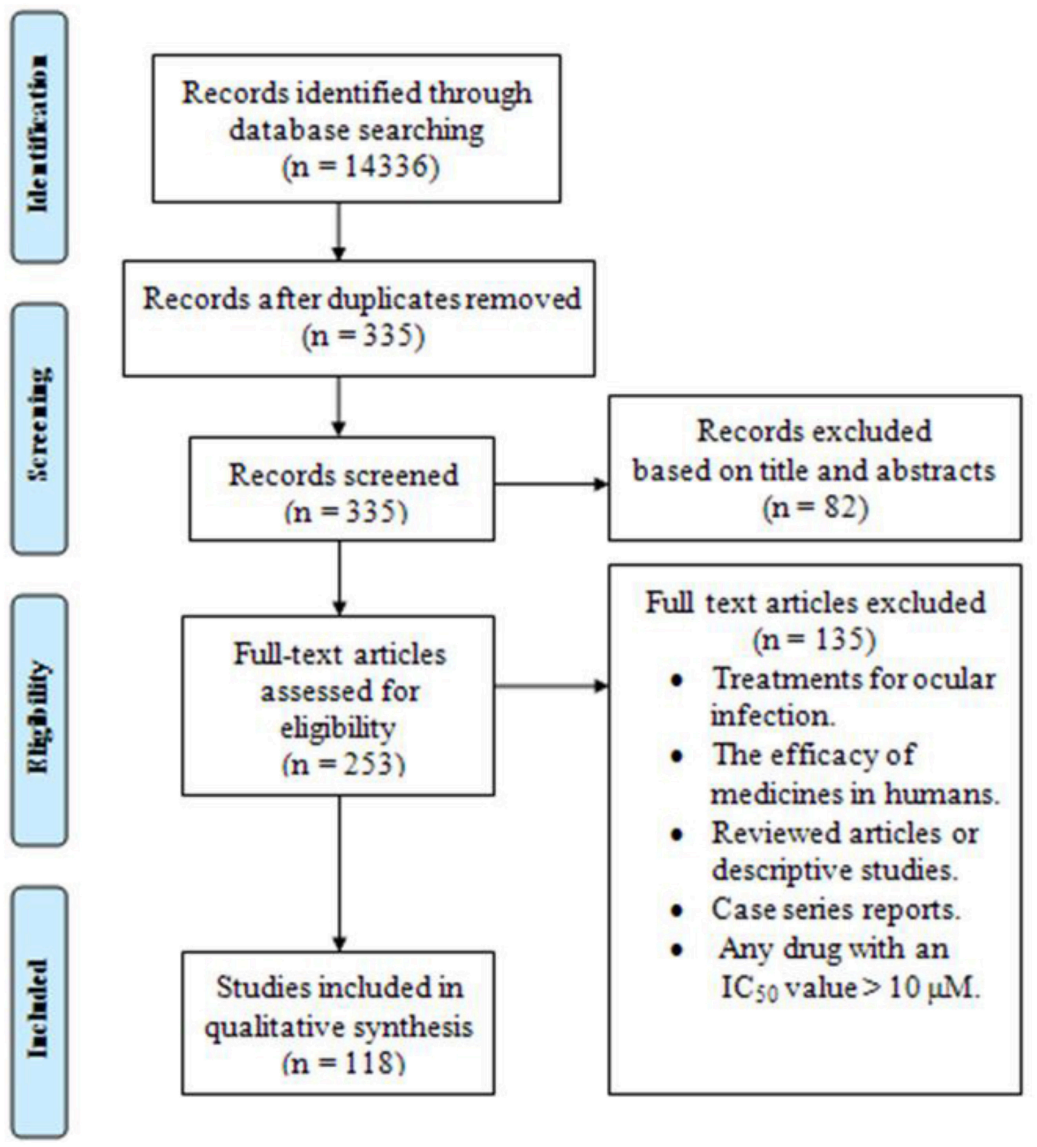
**The PRISMA flow diagram of the search strategy, study selection, and data management procedure of ***in vitro*** and ***in vivo*** activities of anti***-Toxoplasma*** drugs and compounds (2006–2016)**.

A total of 118 papers (83 studies *in vitro*, 59 *in vivo*, 27 both *in vitro* and *in vivo*) published from 2006 to 2016, were included in the systematic review. Figure 1 briefly shows the search process in this systematic review article.

### Mechanisms of action

**Figure 2:**
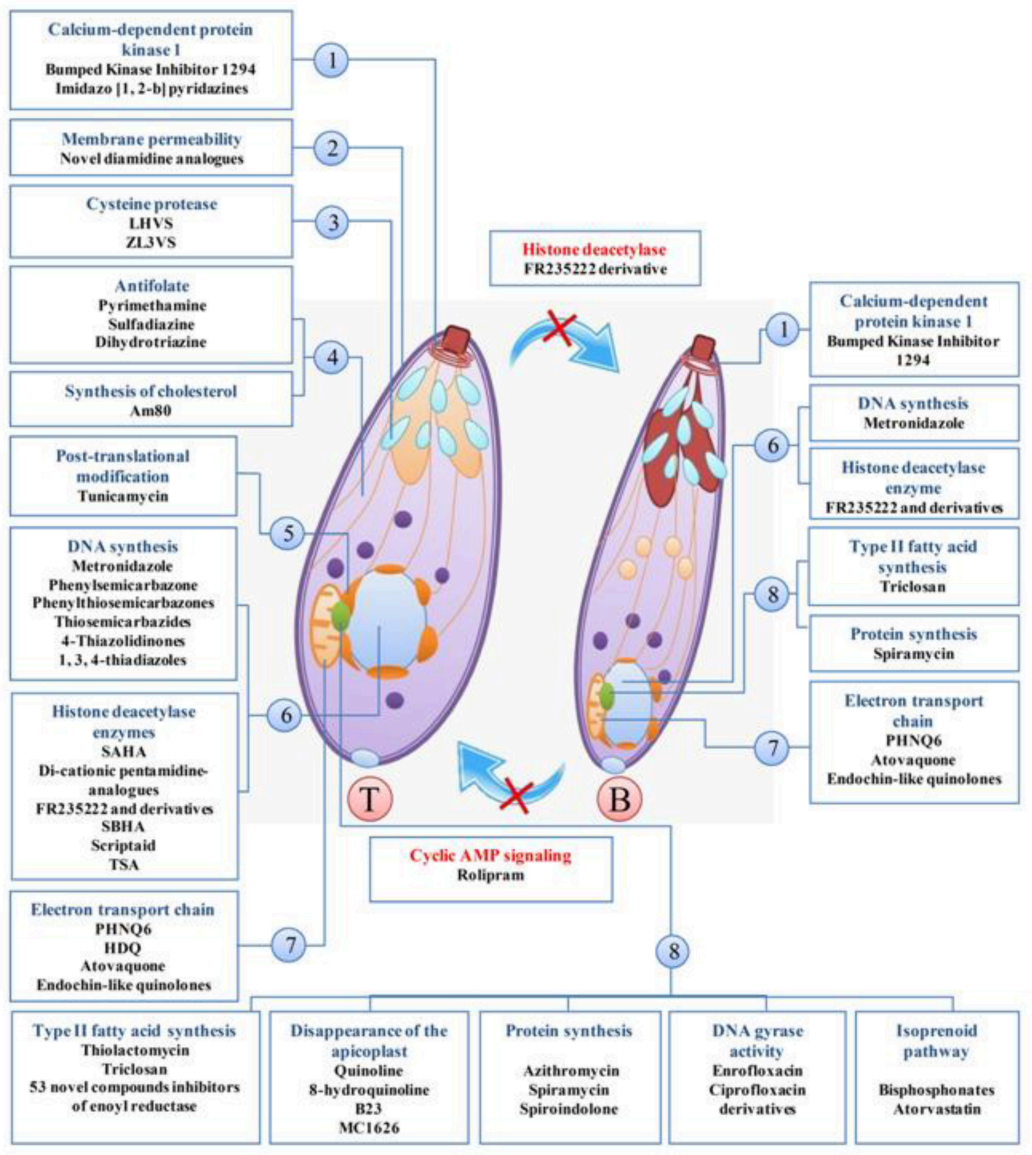
**Drugs/compounds with known mechanisms of action on life stages of ***T. gondii, tachyzoites (T), and bradyzoites*** (B)**. 1, apical end; 2, Cell membrane; 3, microneme; 4, cytosol; 5, endoplasmic reticulum; 6, core; 7, mitochondria; 8, apicoplast.

In the current systematic review, 80 clinically available drugs (Table 1) and several new compounds with more than 39 pathways/ mechanisms of action were evaluated against *T. gondii* in both *in vitro* and *in vivo* studies. Several target based drug screens were also identified against *T. gondii* include mitochondrial electron transport chain, calcium-dependent protein kinase 1, type II fatty acid synthesis, DNA synthesis, DNA replication, etc. (Table 2). Also, drugs/compounds with known mechanisms of action on life stages of *T. gondii* are shown in Figure 2. Our collective data indicated that many of the drugs/ compounds evaluated against *T. gondii* act on the apicoplast. Therefore, the apicoplast represents as a potential drug target for new chemotherapy.

**Table 1:** **Clinically available drugs/compounds evaluated against ***T. gondii in vitro*** and ***in vivo*** studies**.

**Common clinical uses**	**Drugs/compounds**	**References**
Antiprotozoal agents	Bisphosphonates	Baramee et al., 2006; Ferreira et al., 2006; Rajapakse et al., 2007; Strobl et al., 2007; Leepin et al., 2008; Shubar et al., 2008; Liesen et al., 2010; Aquino et al., 2011; Franco et al., 2011; Martins-Duarte et al., 2011; Chew et al., 2012; Asgari et al., 2013; Bilgin et al., 2013; Gomes et al., 2013; Gaafar et al., 2014; da Silva et al., 2015; El-Zawawy et al., 2015a,b
	Diamidine analogs	
	Spiramycin (Rovamycin)	
	Thiosemicarbazides	
	4-thiazolidinones	
	1,3,4-thiadiazoles	
	Naphthalene-sulfonyl-indole	
	Thiosemicarbazone	
	Phenylsemicarbazone	
	Ivermectin	
	Silver nanoparticles	
	Novel ferrocenic atovaquone derivatives	
	Triclosan	
	Triclosan liposomal nanoparticles	
	Metronidazole	
	1,25(OH)2D3	
	Naphthoquinone	
	PHNQ6^a^	
	Novel azasterols	
	Apicidin	
Antimalarial agents	Pyrimethamine	Meneceur et al., 2008; Mui et al., 2008; Doggett et al., 2012; Zhou et al., 2014; Jain et al., 2015
	Atovaquone	
	Triazine JPC-2067-B	
	Spiroindolone	
	Endochin-like quinolones	
	Halofuginone	
Antibacterial agents	Sulfadiazine	Meneceur et al., 2008; Costa et al., 2009; Barbosa et al., 2012; Payne et al., 2013; Castro-Filice et al., 2014; Gaafar et al., 2014; Martins-Duarte et al., 2015
	Azithromycin	
	Enrofloxacin	
	Fusidic acid	
	Ciprofloxacin	
	Chitosan	
Antiretroviral agents	Atazanavir	Monzote et al., 2013
	Fosamprenavir	
	Indinavir	
	Nelfinavir	
	Ritonavir	
	Saquinavir	
Anticoccidial agents	NPPP^b^	Kul et al., 2013; Choi et al., 2014; Oz, 2014a,b
	Diclazuril	
	Toltrazuril	
Antihelminthic agents	Niclosamide	Fomovska et al., 2012; Galván-Ramírez et al., 2013
	Nitazoxanide	
Antifungal agents	Itraconazole	Martins-Duarte Edos et al., 2008; Martins-Duarte et al., 2010, 2013; Gaafar et al., 2014
	Fluconazole	
	Chitosan	
Anticancer agents	SAHA^c^	Strobl et al., 2007; Portes Jde et al., 2012; Leyke et al., 2012; Barna et al., 2013; Kadri et al., 2014; de Lima et al., 2015; Eissa et al., 2015; Opsenica et al., 2015; Dittmar et al., 2016
	Pterocarpanquinone	
	Ruthenium complexes	
	Quinoline derivatives 4-aminoquinoline	
	4-piperazinylquinoline analogs	
	Miltefosine	
	Tetraoxanes	
	Gefitinib	
	3-bromopyruvate	
	Tamoxifen	
Immunosuppressants agents	Auranofin	Ghaffarifar et al., 2006; Wei et al., 2007; Andrade et al., 2014; Ihara and Nishikawa, 2014
	Am80	
	Betamethasone	
	Pyridinylimidazole	
	Imidazopyrimidine	
Immunomodulators agents	Rolipram	Afifi and Al-Rabia, 2015
Immunoregulatory agents	Levamisole	Köksal et al., 2016
Antipsychotic agents	Aripiprazole	Saraei et al., 2015
Antioxidant agents	Resveratrol	Bottari et al., 2015
Antischizophrenic agents	Haloperidol	Goodwin et al., 2011; Fond et al., 2014; Saraei et al., 2016
	Clozapine	
	Fluphenazine	
	Trifluoperazine	
	Thioridazine	
	Amisulpride	
	Cyamemazine	
	Levomepromazine	
	Loxapine	
	Olanzapine	
	Risperidone	
	Tiapride	
Moodstabilizing agents	Valproate	Fond et al., 2014
Anti hypertensive agents	Guanabenz	Benmerzouga et al., 2015
Anti hypertensive and irregular heart rate agents	Propranolol	Montazeri et al., 2015, 2016

a *2-hydroxy-3-(1′-propen-3-phenyl)-1,4-naphthoquinone*.

b *(4-nitrophenoxy) phenyl] propane one*.

c *Suberoylanilide hydroxamic acid*.

**Table 2:** **Drugs/compounds with pathways/ mechanisms of action against ***T. gondii*****.

**Pathway/mechanism of action**	**Drugs/compounds**	**References**
Electron transport chain	PHNQ6^*^^a^	Baramee et al., 2006; Ferreira et al., 2006, 2012; Saleh et al., 2007; Meneceur et al., 2008; Bajohr et al., 2010; Doggett et al., 2012; Kul et al., 2013; de Lima et al., 2015
	HDQ^*^^b^	
	Atovaquone^*^	
	Endochin-like quinolones^*^	
	Ferrocenic atovaquone derivatives	
	Naphthoquinones	
	Toltrazuril	
	3-Bromopyruvate	
Sterol biosynthesis	Novel quinuclidine (ER119884, E5700)	Martins-Duarte et al., 2006
Synthesis of cholesterol	Am80^*^	Ihara and Nishikawa, 2014
Antifolate	Pyrimethamine^*^	Meneceur et al., 2008; Mui et al., 2008; Martins-Duarte et al., 2013
	Sulfadiazine^*^	
	Dihydrotriazine^*^	
	(JPC-2067-B, JPC-2056)	
Calcium-dependent protein kinase 1	1 NM-PP1^*^	Sugi et al., 2011; Doggett et al., 2014; Moine et al., 2015b; Vidadala et al., 2016
	Bumped Kinase Inhibitor 1294^*^	
	Imidazo [1,2-b] pyridazines^*^	
	Compound 32^*^	
Human mitogen-activated protein kinase	Pyridinylimidazole^*^	Wei et al., 2007
	Imidazopyrimidine^*^	
Nucleoside triphosphate hydrolase (NTPase)	2-(Naphthalene-2-γlthiol)-1H indole^*^	Asgari et al., 2013, 2015
Isoprenoid pathway	2- alkylaminoethyl- 1,1- bisphosphonic acids^*^	Shubar et al., 2008; Szajnman et al., 2008; Li et al., 2013
	Newly synthesized bisphosphonates^*^	
	Atorvastatin^*^	
Type II fatty acid synthesis	Thiolactomycin^*^	Martins-Duarte et al., 2009; Tipparaju et al., 2010; El-Zawawy et al., 2015a,b
	53 novel compounds^*^	
	Inhibitors of enoyl reductase	
	Triclosan and triclosan liposomal^*^	
Protein synthesis	Azithromycin^*^	Costa et al., 2009; Franco et al., 2011; Chew et al., 2012; Zhou et al., 2014; Palencia et al., 2016
	Spiramycin^*^	
	Spiroindolone	
	3-aminomethyl benzoxaborole (AN6426)	
Disappearance of the Apicoplast	Quinoline derivatives^*^	Smith et al., 2007; Kadri et al., 2014
	(MC1626, quinoline, 8-hydroquinoline and B23)	
Histone deacetylase enzyme	SAHA^*^^c^	Strobl et al., 2007; Maubon et al., 2010; Kropf et al., 2012
	SBHA^*^^d^	
	Scriptaid^*^	
	Trichostatin A^*^	
	Di-cationic pentamidine-analog^*^	
	FR235222, FR235222 derivative^*^	
DNA synthesis	Metronidazole^*^	Liesen et al., 2010; Chew et al., 2012; Gomes et al., 2013
	Phenylsemicarbazone^*^	
	Phenylthiosemicarbazones^*^	
	Thiosemicarbazides^*^	
	4-Thiazolidinones^*^	
	1,3,4-thiadiazoles^*^	
Cyclic AMP signaling pathways	Rolipram^*^	Afifi et al., 2014; Afifi and Al-Rabia, 2015
Post-translational modification by N-linked glycosylation of proteins	Tunicamycin^*^	Luk et al., 2008
Membrane permeability	Novel diamidine analog^*^	Leepin et al., 2008
Microfilament functional	Cromolyn sodium	Endeshaw et al., 2010; Rezaei et al., 2014; Montazeri et al., 2015, 2016
	Ketotifen	
	Propranolol	
	Oryzalin analogs	
Micronemal secretion pathway, cysteine protease	Peptidyl vinyl sulfone compounds^*^ **(**LHVS and ZL3VS**)**	Teo et al., 2007
Immuno-regulatory	Levamisole^*^	Köksal et al., 2016
Translational control	Guanabenz^*^	Payne et al., 2013; Benmerzouga et al., 2015; Jain et al., 2015
	Fusidic acid	
	Halofuginone^*^	
DNA gyrase activity, transcription	Enrofloxacin	Barbosa et al., 2012; Martins-Duarte et al., 2015
	Ciprofloxacin derivatives^*^	
Thioredoxin reductase	Auranofin	Andrade et al., 2014
Topoisomerases I and II HSP90 protein	Harmane, norharmane, and harmine	Alomar et al., 2013
Metabolism of neurotransmitters in the brain	Resveratrol	Bottari et al., 2015
Effect on the liver biochemical parameters	ATT-5126 and KH-0562	Choi et al., 2014
Vascular ATP synthase subunit C and/or methyltransferase	NPPP	Choi et al., 2015
Sterol biosynthesis enzyme-sterol methyl transferase.	22, 26-azasterol and 24, 25-(R, S)- epiminolanosterol	Martins-Duarte et al., 2011
Downregulates expression of serine/threonine protein phosphatase	Diclazuril	Oz, 2014a,b
Ergosterol synthesis	Fluconazole	Martins-Duarte Edos et al., 2008; Martins-Duarte et al., 2013
	Itraconazole	
Interruption of mitosis	Trifluralin	Wiengcharoen et al., 2007
Oxidative phosphorylation	Niclosamide	Fomovska et al., 2012
Apocynin-dependent pathway	NSC3852	Strobl et al., 2009
Phospholipid metabolism	Miltefosine	Eissa et al., 2015
Quinone oxidoreductase expression	Nitaxozanide	Galván-Ramírez et al., 2013
Kinase inhibitors	Small-molecules	Kamau et al., 2012
Tyrosine kinase	Gefitinib	Yang et al., 2014
	Crizotinib	
Adenosine kinase in the purine salvage pathways	N6-benzyladenosine analog^*^	Kim et al., 2007; Szajnman et al., 2008
Purine nucleoside phosphorylase	3-(thiophen-2-yl)-1,2,4-triazole-5-thione	Dzitko et al., 2014b
Damage on the microneme proteins	7-nitroquinoxalin-2-ones (VAM2-2)	Fernández et al., 2016

* *Drugs/compounds with known pathway/mechanisms of action gainst T. gondii*.

a *2-hydroxy-3-(1′-propen-3-phenyl)-1,4-naphthoquinone*.

b *1-hydroxy-2-dodecyl-4 (1H) quinolone*.

c *Suberoylanilide hydroxamic acid*.

d *Suberic bishydroxamic acid*.

### The investigated strains

*T. gondii* has three main clonal lineages in population structure; type I (including a highly virulent RH strain), Type II (including ME49 and PRU, avirulent strains), and Type III (including avirulent strains like NED), which is correlated with virulence expression in mice (Howe and Sibley, 1995).

*In vitro* and *in vivo* screening methods were used of type I *T. gondii* (mostly RH strain; 76 studies *in vitro*, and 36 *in vivo*). Because type I RH strain is highly virulent in mice, causing 100% mortality, but types II and III are relatively less virulent. Although in some studies, ME49 (7 studies *in vitro*, and 17 *in vivo*), Prugniaud, EGS, and VEG strains were used, which showed that the outcome of infections depends on the challenge dose and on the genotype of the host (Szabo and Finney, 2016). Details about the investigated strains *in vitro* and *in vivo* are shown in Tables 3, 4, respectively.

**Table 3:** **Summary of ***in vitro*** studies evaluated the anti-***Toxoplasma*** activity of drugs/compounds**.

**No**	**Drug**	**Strain**	**Cells**	**Culture**	**Evaluation**	**Main results**	**Effectivity**	**Positive control**	**References**
1	Two novel quinuclidine (ER119884, E5700)	RH	LLCMK2	24, 48 h	IC_50_ values^a^	IC_50_ ER119884, E5700 = 0.66, 0.23 μM	Effective	Sulfadiazine, pyrimethamine	Martins-Duarte et al., 2006
2	Fourteen novel ferrocenic atovaquone derivatives	76K, PLK, A to R	HFF	48 h	IC_50_ values	IC_50_ 2d, 2e, 2f = 5.0, 2.5, 6.25 μM	Effective 2d, 2e, 2f	–	Baramee et al., 2006
3	Betamethasone and IFN-γ^b^	RH	Hela	24, 48, 72 h	Counting the number of tachyzoites	High number of plaques was seen in group with 40 μg/ml of betamethasone.	Betamethasone not effective, IFN–γ effective	–	Ghaffarifar et al., 2006
4	Suberoylanilide hydroxamic, suberic bishydroxamic acid, scriptaid, trichostatin A	RH	HS68 HFF	48, 72 h	IC_50_ values	IC_50_ scriptaid = 0.039 μM	Scriptaid was the most effective	–	Strobl et al., 2007
5	RWJ67657, RWJ64809^c^, RWJ68198^d^	RH, ME49	HFF	48 h	IC_50_ values	RWJ67657 was at least as potent as RWJ68198, SB203580, or SB202190 in reducing of *T. gondii* replication	RWJ67657, SB203580 effective	–	Wei et al., 2007
6	Novel drug compounds (A–I) (B,F,G,H) (trifluralin analogs)	RH	Vero	72 h	MTT assay^e^, crystal violet assay	IC_50_ drug F = 10 μM	Drugs F was the most effective	–	Wiengcharoen et al., 2007
7	1-hydroxy-2-dodecyl-4(1H) quinolone (HDQ)	RH	HFF	24 h	Replication rate determined	IC_50_ HDQ = 0.0024 ± 0.0003 μM	Effective	–	Saleh et al., 2007
8	Quinoline derivative MC1626	RH	HFF	24 h	Standard [3H]uracil uptake and plaque assays	100 μM reducing growth	Effective	–	Smith et al., 2007
9	N6-benzyladenosine analogs	RH	HFF	24 h	MTT assay	IC_50_ N6-(2,4-dimethoxybenzyl) Adenosine = 8.7 ± 0.6 μM, exhibited the most favorable activity	Effective	Sulfadiazine, pyrimethamine	Kim et al., 2007
10	Fluorine-containing aryloxyethyl thiocyanate derivatives	RH	HFF	24 h	IC_50_ values	IC_50_ compounds 1 and 3 = 2.80 and 3.99 μM	Effective	Atovaquone	Liñares et al., 2007
11	LHVS, ZL3VS^f^	RH or 2F1	HFF	45 min	B gal^g^, Red/green invasion assay, SDS-PAGE, immunoblotting, gliding motility assay	IC_50_ LHVS and ZL3 VS = 10 and 12.5 μM	Effective	3,4-dichloroisocoumarin	Teo et al., 2007
12	1,25(OH) 2D3	RH	MICc12	72 h	Trypan blue assay	Ruled out any toxic effects of 1,25(OH) 2D 3 for *T. gondii*	Effective	–	Rajapakse et al., 2007
13	Tunicamycin	RH	HFF	2, 24, or 48 h	Fluorescence and electron microscopy	N-Glycosylation is completely inhibited by treatment of parasites with tunicamycin	Effective	Pyrimethamine	Luk et al., 2008
14	Novel diamidine analogs	RH	Vero HFF	2 or 3 days	IC_50_ values, Q-PCR^h^	IC_50_ DB750, DB786 = 0.16, 0.22 μM	Effective	–	Leepin et al., 2008
15	Pyrimethamine, sulfadiazine, and atovaquone	17 strains *T. gondii*	THP-1 MRC-5	7 days	IC_50_, real-time PCR	IC_50_ pyrimethamine = 0.0002, 0.01 μM	Effective	–	Meneceur et al., 2008
						IC_50_ atovaquone = 0.0001, 0.00005 μM			
						IC_50_ sulfadiazine = 0.01, 0.07 μM for			
						13 strains and were > 0.1 μM for three strains			
16	Novel triazine JPC-2067-B	RH	HFF	3 days	Liquid scintillation counting	IC_50_ JPC-2067-B = 0.02 μM,	Effective	–	Mui et al., 2008
						IC_90_ JPC-2067-B = 0.05 μM			
17	Newly synthesized bisphosphonates (15 new compounds)	RH	Mouse macrophages (J 744A.1)	24, 48 h	MTT assay, flow cytometry	91A and 282A showed moderate and low toxicity (cell viability between 70% and 100%)	Effective	–	Shubar et al., 2008
18	2-alkylaminoethyl- 1,1-bisphosphonic acids	RH	HFF	Daily	IC_50_ values, radiometric assay	IC_50_ compound 19 = 2.6 μM	Compound 19 was very effective	.	Szajnman et al., 2008
19	Itraconazole	RH	LLCMK2	24 or 48 h	IC_50_ values, TEM^i^ analysis	IC_50_ = 0.11, 0.05 μM for 24, 48 h	Effective	–	Martins-Duarte Edos et al., 2008
20	Thiolactomycin analogs (8 new compounds)	RH	LLCMK2	24, 48 h	IC_50_ values, Lipid extraction, chromatographic analysis	IC_50_ compounds = 1.6-29.4 μM	Compound 5 was very effective	Sulfadiazine, pyrimethamine	Martins-Duarte et al., 2009
21	NSC3852^j^	RH	HS 68 HFF	2 h	SYBR green assay, MTS assay, ROS assay, NO assays	EC_50_ NSC3852 = 0.08 μM,	NSC3852, NSC74949 were the most effective	–	Strobl et al., 2009
						EC_50_ NSC74949 = 0.6 μM			
22	FR235222, FR235222 derivative compounds (W363, W371, W399, W406, W425)	RH, PRU (type II)	HFF	24 h	EC50 determination, Western blot analysis, immunofluorescence microscopy	100% altered cysts 24 h after treatment with the lowest concentration of FR235222	Effective	–	Maubon et al., 2010
23	Thiosemicarbazides, 4-thiazolidinones and 1,3,4-thiadiazoles	RH	Vero	24 h	Mean number of intracellular parasitesa, LD_50_^k^	A significant decrease in the percentage of infected cells and in the mean number of tachyzoites per cell from the concentrations of 0.1, 1, 10 mM	Effective	Hydroxyurea, sulfadiazine	Liesen et al., 2010
24	FLZ^l^ and ITZ^m^	RH	LLCMK2	24, 48 h	IC_50_ values	IC_50_ FLZ = 8.9, 3.1 μM after 24, 48 h	Effective	Sulfadiazine, pyrimethamine	Martins-Duarte et al., 2010
						IC_50_ ITZ = 0.1, 0.05 μM for 24, 48 h			
25	1-Hydroxy-2-Alkyl-4(1H) Quinolone Derivatives	RH (type I)	HFF	24 h	IC_50_ values	IC_50_ compound A, B = 0.0004, 0.0008 μM	Effective	Atovaquone	Bajohr et al., 2010
26	Oryzalin Analogs	RH	HFF	8 day 26 h	Plaque assay, Immunofluorescence assay, IC_50_ values	IC_50_ 18b = 0.03 μM	Effective	–	Endeshaw et al., 2010
27	53 novel compounds (Inhibitors of Enoyl reductase)	RH	HFF	3 days	IC_50_ values	IC_50_ compounds 2, 19 = 0.04, 0.02 μM,	Compounds 2, 19, 39 greatest effect	–	Tipparaju et al., 2010
						IC_50_ compounds 39 less active			
28	Haloperidol, clozapine, fluphenazine, trifluoperazine, thioridazine	RH	HFF	48 h	IC_50_ values	IC_50_ fluphenazine, thioridazine, trifluoperazine = 1, 1.2, and 3.8 μM	Fluphenazine, thioridazine, trifluoperazine were effective	–	Goodwin et al., 2011
29	Azithromycin, spiramycin	RH	Bewo cell line	24 h	MTT assay, measurement of Th1/Th2	Increase TNF-a^n^, IL-10, IL-4 production, but decreased IFN-γ	Effective	–	Franco et al., 2011
30	Novel azasterols	RH ME49	LLCMK2	24 or 48 h	IC_50_ values, imunofluorescence assays	IC_50_ compounds 1, 2, 3 = 0.8–4.7 μM	Compound 3 was the most effective	–	Martins-Duarte et al., 2011
31	Ciprofloxacin derivatives	RH	LLC-MK2	24 or 48 h	IC_50_, MTS assay	IC_50_ compounds 2, 4, 5= 0.42, 1.24, and 0.46 μM	Effective	–	Dubar et al., 2011
32	2-hydrazolyl-3-phenyl-5-(4-nitrobenzylidene)-4-thiazolidinone substituted	RH	Vero	24 h	LD_50_ values	LD_50_ = 0.5, 10 mM	Effective	Hydroxyurea, Sulfadiazine	Aquino et al., 2011
33	Nanoparticles	RH (CAT-GFP)	Macrophages J 774-A1	3 day	HPLC^o^: flow cytometry	Ca^p^.85% observed maximum in Toxoplasmosis therapy efficiency	Effective	–	Leyke et al., 2012
34	Enrofloxacin	RH	HFF	72 h	MTT assays	Enrofloxacin resulted in a significant inhibition of the percentage of infected cells by the parasite (58.72%)	Effective	Sulfadiazine, pyrimethamine	Barbosa et al., 2012
35	ELQ-271 and ELQ-316^q^	2F	HFF	4 days	Host-cell toxicity	IC_50_ ELQ-271, ELQ-316 = 0.0001, and 0.000007 μM	Effective	Atovaquone	Doggett et al., 2012
36	Pterocarpanquinone	RH	LLCMK2	24 or 48 h	Direct counts, viability, imunofluorescence assays	IC_50_= 2.5 μM	Effective	–	Portes Jde et al., 2012
37	New naphthoquinones and an alkaloid	RH, EGS	HFF	48 h	MTT assays	IC_50_ QUI-5, and QUI-6^r^ = 69.35, and 172.81 μM	Effective	Atovaquone, Sulfadiazine	Ferreira et al., 2012
38	Spiramycin coadministered with metronidazole	ME49	Vero E6	1 week	Numbers of cysts and tachyzoites	Spiramycin reduced *in vitro* reactivation, metronidazole alone did not have significant effect	Effective	–	Chew et al., 2012
39	Di-cationic pentamidine-analogs	RH ME49	HFF	72 h	Cytotoxicity assays	IC_50_ arylimidamide DB745 = 0.11, 0.13 μM (tachyzoites of Rh, Me49)	Effective	Atovaquone	Kropf et al., 2012
40	Small-Molecule (*n*=527)	Strains 5A10 (type III strain)	HFF	72 h	Luciferasebased assay, Host cell viability, electron microscopy, invasion, motility assays	EC_50_ s for the 14 compounds = 0.14–8.7 μM	14 compounds effect	–	Kamau et al., 2012
41	Salicylic acids (39 compounds)	RH, RH-YFP, and ME49	HFF	1 h	[3H]-Uracil incorporation and YFP Fluorescence assay	3i, 3j, 7a, 14a, and 14b were active at low nanomolar concentrations	Effective	Pyrimethamine, Sulfadiazine	Fomovska et al., 2012
42	FLZ combined with sulfadiazine and pyrimethamine	RH	LLCMK2	24 h	IC50 values and MTS assay	IC_50_ FLZ = 8.4 ± 1.2, IC_50_ sulfadiazine/pyrimethamine, pyrimethamine = 8.7 ± 0.8 μM	Effective	–	Martins-Duarte et al., 2013
43	Harmane, Norharmane (β-carboline alkaloids)	RH	Vero HFF	1, 24 h	Parasite invasion and replication rate	harmane and harmine showed 2.5- to 3.5-fold decrease in the invasion rates at doses of 40 μM, norharmane 2.5 μM	Effective	Sulfadiazine	Alomar et al., 2013
44	Fusidic acid	Prugniaud	HFF	7 days	Lytic plaques counted	IC_50_ = 7.7 μM, decreased the number of *T. gondii* plaques in a dosedependent manner	Effective	–	Payne et al., 2013
45	Two naphthalene-sulfonyl-indole compounds	RH	–	1.5 h	Stained by PI, analyzed by FACS	LD_50_ compound A, B = 62, 800 μmol	Effective	Saponin	Asgari et al., 2013
46	(Benzaldehyde)-4-phenyl-3- thiosemicarbazone, (benzaldehyde)-(4 or 1)- phenylsemicarbazone (9 compounds)	RH	Vero	24 h	Cytotoxicity, number of intracellular parasites	LD_50_ compound 8 = 0.3 mM, reduced the number of intracellular parasites by 82 % in a concentration of 0.01 mM	Effective	Sulfadizine	Gomes et al., 2013
47	Ivermectin and sulphadiazine	RH	Hep- 2	24, 48, 72 h	IC_50_, invert microscopy, ELISA assay	IC_50_ ivermectin and sulphadiazine = 0.2, and 29.1 μM	Effective	–	Bilgin et al., 2013
48	Novel ruthenium complexes	RH	HFF	72 h	cytotoxicity assessment, TEM	EC_50_ compounds 16, 18 = 18.7, 41.1 nM	Compounds 16, and 18 effective	–	Barna et al., 2013
49	Atazanavir, fosamprenavir, indinavir, nelfinavir, ritonavir, and saquinavir	RH	Macrophages Swiss Webster	48 h	IC_50_ determination, MTT assay	IC_50_ atazanavir ritonavir, and saquinavir = > 1 μM	Effective	Pyrimethamine	Monzote et al., 2013
						IC_50_ fosamprenavir, and nelfinavir = > 5 μM			
50	Atorvastatin	RH	HFF	8 days	IC_50_ values	IC_50_ = 50 μM	Effective	–	Li et al., 2013
51	Nitaxozanide	RH	Astrocyte	24,48 h	Immunocytochemical method, microscopic analysis, viability	Nitazoxanide produced 97% *T. gondii* death in a concentration of 10 mg/mL in 48 h infected astrocytes	Effective	Pyrimethamine	Galván-Ramírez et al., 2013
52	Amisulpride, cyamemazine, fluphenazine, haloperidol, levomepromazine, loxapine, olanzapine, risperidone, tiapride, and valproate	RH	HFF	4 h	Growth inhibition assay	Amisulpride, tiapride and valproate did not have inhibitory activity	Zuclopenthixol, high effective	–	Fond et al., 2014
53	Spiroindolone	RH	HFF	72 h	Fluorescence assays, cytotoxicity assessment	IC_50_ = 1 μM	Effective	Pyrimethamine, sulfadiazine	Zhou et al., 2014
54	Auranofin	RH	HFF	5 days	Invasion and replication assays and plaque assays	TD_50_ = 8.21 μM, IC_50_ = 0.28 μM	Effective	Pyrimethamine, Sulfadiazine	Andrade et al., 2014
55	Azithromycin	2 F1	Placental tissues	48 h	Production of cytokines and hormones	Increases IL-6 production, reduced secretion of estradiol, progesterone, and HCG + β	Effective	Pyrimethamine, Sulfadiazine, folinic acid	Castro-Filice et al., 2014
56	6-Trifluoromethyl-2-thiouracil (ATT-5126), (KH-0562)	RH	Hela	24 h	MTS assay, IC_50_	IC_50_ ATT-5126, KH-0562 = 19.7, 32.2 μM	Effective	Pyrimethamine	Choi et al., 2014
						CC_50_ ATT-5126, KH-0562 = 35.4, 56.3 μM			
57	Cromolyn sodium and ketotifen	RH	Macrophage monolayer	24 h	Inhibition rate	After 60 min the best efficacy was observed at 15 μg/ml (78.9 ± 1.70, 91.97 ± 0.37%)	Effective	–	Rezaei et al., 2014
58	200 drug-like and 200 probe-like compounds of Malaria Box	TS-4 (mutant of the RH)	HFF	24 h	Cytotoxicity assays	Seven compounds with IC_50_ < 5 μM, SI > 6	7 compounds effected	Pyrimethamine, sulfadiazine	Boyom et al., 2014
59	Am80	RH, PLK, its recombinants	J 774A.1	20 h	Uracil incorporation assay, RT–PCR^s^, flow cytometry	Am80 inhibited parasite growth by decreasing intracellular accumulation of cholesterol	Effective	–	Ihara and Nishikawa, 2014
60	Pyrimethamine –loaded lipid-core nanocapsules	RH	LLC-MK2	72 h	MTS assay	TC_50_ PYR loaded lipid-core nanocapsules = 6.0 μM	Effective	–	Pissinate et al., 2014
61	Quinoline derivatives (58 compounds)	2F	HFF	4 days	Cytotoxicity assays	IC_50_ B23 = 0.4 ± 0.03 μM, the most effective compound	32 compounds effected	–	Kadri et al., 2014
62	74 novel thiazolidin-4-one derivatives	–	HFF	5 days	Cytotoxicity assays	IC_50_ derivatives 12 A, 27 A = 0.9, 2.9 μM	Effective	Timethoprim	D'Ascenzio et al., 2014
63	Gefitinib and Crizotinib	RH	Hela	24, 48, 72 h	Counting the number of *T. gondii* per parasitophorous vacuolar membrane	Gefitinib inhibited the growth of *T. gondii* over 5 μM whereas Sunitinib did not	Gefitinib effected	Pyrimethamine	Yang et al., 2014
64	1,4-disubstituted thiosemicarbazides	RH	Mouse L929 fibroblasts	24 h	MTT assay and q-pcr	1g, 2b, 3d, 3l showed significant anti-parasitic effects	1 g was very effective	Sulfadiazine	Dzitko et al., 2014a
65	3-(thiophen-2-yl)-1,2,4-triazole-5-thione	RH	Mouse L929 fibroblasts	24 h	IC_50_ values and q-pcr	IC_50_ at least 30 times better than that of sulfadiazine	Effective	Sulfadiazine	Dzitko et al., 2014b
66	1-[4-(4-nitrophenoxy) phenyl]propane-1-one (NPPP)	RH	Hela	24 h	CC_50_, EC_50_ values	EC_50_, CC_50_ = 36.2 ± 0.2, 67.0 ± 0.2 μM	Effective	–	Choi et al., 2015
67	C-type lectin from Bothropspauloensis venom	RH	Hela	24 h	MTT assay, cytokine measurements	MTT assay between 0.195, 12.5 μg/mL MIF, IL-6 productions were increased	Effective	–	Castanheira et al., 2015
68	Ciprofloxacin derivatives Compounds (2, 4, 5)	RH	LLCMK2 HFF	24, 48, 72 h	Immunofluorescence, TEM	Inhibited parasite replication early in the first cycle of infection	Effective	–	Martins-Duarte et al., 2015
				6 h					
69	New chiral N-cylsulfonamide bis-oxazolidin-2-ones	RH	MRC-5	–	IC_50_ values	IC_50_ of Mol 1 was less than Mol 2	Effective	Sulfadiazine	Meriem et al., 2015
70	Guanabenz	ME49 Prugniaud	HFF	32 h	EC_50_ values	EC_50_ = 6 μM	Effective	–	Benmerzouga et al., 2015
71	3-Bromopyruvate, Atovaquone	RH	LLC-MK2	24, 48 h, or 6 days	Light-microscopic analysis, indirect immunofluorescent assays	73 and 71% reduction in intracellular parasites after 24, 48 h	Effective	–	de Lima et al., 2015
72	Biphenylimidazoazines	RH	HFF	96 h	EC_50_ values and fluorescence microscopy assay	EC_50_ < 1 μM	Effective	Pyrimethamine	Moine et al., 2015a
73	Halofuginone	RH	HFF	24 h	EC_50_ values	EC_50_ = 0.94 nM	Effective	Pyrimethamine	Jain et al., 2015
74	Naphthoquinone derivative	RH	LLC-MK2	24, 48 h	IC_50_ and MTT assay	IC_50_ LQB 151 = < 1 μM	Effective	–	da Silva et al., 2015
75	Aryloxyethyl thiocyanates	RH	Vero	24 h	Determination of ED_50_	ED_50_ derivatives 15 and 16 = 1.6 μM and 1.9 μM	Effective	–	Chao et al., 2015
76	Imidazo [1,2-b] pyridazines derivatives	RH-GFP	HFF	24 h	Cytotoxicity assay	EC_50_ 16a, 16f = 100, 70 nM	Effective	–	Moine et al., 2015b
77	Nitrofurantoin	RH	Hela	24 h	MTS assay	Selectivity = 2.3	Effective	Pyrimethamine	Yeo et al., 2016
						EC_50_ = 14.7 μM			
78	Quinoxalinone derivatives	RH	HEp-2^t^	24 h	IC_50_ values, viability, invasion, and intracellular growth	MIC_50_ VAM2-2 = 3.3 ± 1.8 μM	VAM2-2 was very effective	–	Fernández et al., 2016
79	1120 compounds	RH-GFP	HFF	72 h	Parasite invasion, Microneme secretion, Luciferase, and LC3-GFP assays	94 compounds with IC50 < 5 μM	Tamoxifen effective	–	Dittmar et al., 2016
80	3-aminomethyl benzoxaborole (AN6426)	RH	HFF	24 h	Determination of EC_50_	EC_50_ = 76.9 μM	Effective	Pyrimethamine	Palencia et al., 2016
81	Sulfur-containing linear bisphosphonates	RH, Prugniard	Human fibroblasts (hTert cells)	5 days	Determination of EC_50_	EC_50_ = 0.11 ± 0.02 μM	Compound 22 was very effective	–	Szajnman et al., 2016
82	Fluorine-containing Analogs of WC-9 (4-phenoxyphenoxyethyl thiocyanate)	RH	Vero	24 h	Determination of EC_50_	EC_50_ 3-(3-fluorophenoxy), 3-(4-fluorophenoxy) phenoxyethyl thiocyanates, and 2-[3-(phenoxy)phenoxyethylthio]ethyl-1,1-bisphosphonat = 1.6 4.9 and 0.7 μM	Effective	–	Chao et al., 2016
83	6-(1,2,6,7-tetraoxaspiro[7.11] nonadec-4-yl)hexan-1-ol (N-251)	RH	Human hepatocyte, Huh-7	72 h	IC_50_ values, q-pcr, ultrastructural Change by TEM	LC_50_ = 1.11 μg/ml	Effective	Sulfadiazine	Xin et al., 2016

a *Half maximal inhibitory concentration*.

b *Interferon gamma*.

c *Pyridinylimidazole*.

d *Imidazopyrimidine*.

e *3− (4, 5−Dimethyl−2−Thiazyl) − 2, 5−Diphenyl−2H−Tetrazoliu Bromide*.

f *Morpholinourea-leucyl-homophenolalaninyl-phenyl-vinylsulfone, N-benzoxycarbonyl-(leucyl) 3-phenyl-vinyl-sulfone*.

g *B galactosidase*.

h *Quantitative polymerase chain reaction*.

i *Transmission electron microscopy*.

j *5- nitroso-8-quinolinol*.

k *Lethal Dose, 50%*.

l *Fluconazole*.

m *Itraconazole*.

n *Tumor necrosis factor*.

o *High Performance Liquid Chromatography*.

p *Carriers achieved*.

q *Endochin-like quinolones*.

r *7-(4-methyl-3-pentenyl)-2-pyrrolidine-[1, 4]-naphthoquinone (QUI-5), 6-(4-methyl-3-pentenyl)-2-pyrrolidine-[1, 4]-naphthoquinone (QUI-6)*.

s *Reverse transcription polymerase chain reaction*.

t *Human larynx epidermoid carcinoma epithelial cells*.

**Table 4:** **Summary of ***in vivo*** studies evaluated the anti-***Toxoplasma*** activity of drugs/ compounds**.

**No**	**Drug**	**Animal**	**Strain**	**Type of infection**	**Inoculum**	**Treatment**	**Assessment of efficacy**	**Main results**	**Effectivity**	**Positive control**	**References**
1	PHNQ6^a^ alone or combined with sulfadiazine	Female Swiss mice	RH EGS P	Acute, chronic	1000 tachyzoites (ip) 10 brain cysts (orally)	PHNQ6 50 mg/kg/day Sulfadiazine, 40 mg/L	Survival rates, IFAT^b^, and liver histology	Treatment protected at least 70, 90% of mice infected with RH and EGS strains	Effective	Sulfadiazine	Ferreira et al., 2006
2	1, 25(OH) 2D3	BALB/c	ME49	Acute	20 cysts	0.5 μg/kg/2 days ip	Histopathology, RT-PCR^c^	Low parasitic burdens were found	Effective	–	Rajapakse et al., 2007
3	Pyridinylimidazole (RWJ67657, RWJ64809), imidazopyrimidine (RWJ68198)	Female CBA/J, CD8 / –	RH ME49	Acute	1000, 100, and 20 tachyzoites	3.8, 7.5, 15, 30, or 60 mg/kg i.p	Survival rates	The highest dose (60 mg/kg) significantly improved survival	RWJ67657 effective	–	Wei et al., 2007
4	Novel triazine JPC-2067-B	Outbred Swiss Webster	RH	Acute	10000 tachyzoites i.p	1.25 mg/kg/day orally	Peritoneal *T. gondii* burden	Intraperitoneal parasite numbers were reduced	Effective	–	Mui et al., 2008
5	Newly synthesized bisphosphonates	NMRI	RH	Acute	100 000 tachyzoites i.p	490, 1000, 512, 44.05, and 47.6 μM	Flow cytometry	Therapeutic efficacy was 100% for bisphosphonates 2F, 3B, 18A, 22A, and 30B	Effective	–	Shubar et al., 2008
6	Azithromycin, Artemisia annua, spiramycin, SPFA	Females C. callosus	ME49	Chronic	20 cysts	Azithromycin (9 mg/24 h), A. annua (1.0 mg/8 h), spiramycin (0.15 mg/8 h)	Morphological, immunohistochemical analyses, mouse bioassay, and PCR^d^	No morphological changes were seen in the placenta and embryonic tissues from females treated with azithromycin, spiramycin, and SPFA	Azithromycin more effective	–	Costa et al., 2009
7	Dihydroartemisinin and azithromycin	Kunming mice	.	Acute	2 × 10^3^tachyzoites	Dihydroartemisinin and azithromycin 75 and 200 mg/kg	The ultrastructure of tachyzoites	The ultrastructure of tachyzoites was observed in the treatment groups such as edema, enlarged, broken or damaged	Effective	–	Yin et al., 2009
8	FLZ^f^ and ITZ^g^	Outbred female Swiss	CF1 ME49	Chronic	20 cysts of the ME_49_ orally or i.p	10,20 mg/kg/day orally	Survival rates and brain cyst burden	ITZ survival of 90, 87% FLZ survival rate of 71, 85%	Effective	Sulfadiazine, pyrimethamine	Martins-Duarte et al., 2010
9	HDQ^h^ derivatives	Female NMRI, IRF-8 /–	RH ME49	Acute, chronic	10^5^ green fluorescent protein, i.p 10 cysts	32 mg/kg body weight/day	Parasite loads in lungs, livers by qPCR^e^, and flow cytometry analyses	Derivatives of HDQ had lower parasite concentrations than mice treated with HDQ	Effective	Atovaquone	Bajohr et al., 2010
10	FR235222, FR235222 derivative, (W363, W371, W399, W406, W425)	Outbred female Swiss	PRU	Chronic	Living cysts i.p	200 nM	Presence or absence of cysts in brain was assessed by staining	No cysts were detected in mice inoculated with FR235222-treated	Effective	Pyrimethamine	Maubon et al., 2010
11	Azithromycin combined with metronidazole	BALB/c	–	Acutly	50 tissue cysts orally or i.p	250, 200 mg/kg/day	Microscopical examination, bioassay were done for brain, and survival rates	Cure rate 100%	Effective	–	H.Al-jader and Al-Mukhtar, 2010
12	Novel compounds 2,19 (Inhibitors of Enoyl Reductase)	CD1	RH	Acute	2000	10 mg/kg i.p	Parasite burdens in the peritoneal cavity and survival rates	Reduction of parasite burden	Effective	–	Tipparaju et al., 2010
13	SDS-coated atovaquone	C57BL/6	ME49	Acute, chronic	10 cysts orally	100 mg/kg	Histology, PCR	Parasite loads and inflammatory changes in brains were significantly reduced	Effective	–	Shubar et al., 2011
14	1NM-PP1	Old female ICR strain	RH	Acute	1.0 × 10^5^ tachyzoites i.p	5 μM orally	Survival rates, parasite load by qPCR	Reduced the parasite load in the brains, livers, lungs	Effective	–	Sugi et al., 2011
15	Enrofloxacin	Calomys callosus, C57BL/6	RH ME49	Acute, chronic	100 tachyzoites RH strain 20 cysts per 100/ l (orally)	Subcutaneously for 3 days, 3 mg/kg twice a week for the duration of 25-day	Histological analysis, immunohistochemical assay, survival, cyst counts	diminished significantly the tissue parasitism as well as the inflammatory alterations in the brain	Effective	Sulfadiazine, pyrimethamine	Barbosa et al., 2012
16	Small-Molecule (C1, C2, C3, C5)	BALB/c	5A10, PB3-10	Acute, chronic	10,000 tachyzoites i.p	4.4 mg/kg/day	Survival rates, recording the total number of photons per second from each mouse	C2 showed a significant reduction in parasite load in acute and reduced levels of parasite proliferation and increased survival in chronic phase	C2 effective	–	Kamau et al., 2012
17	Endochin-like quinolones (ELQ-271, ELQ-316)	Female CF-1 CBA/J	RH ME49	Acute, chronic	20000 tachyzoites (express YFP) i.p 18 cysts of ME49	50, 20, 5, 1 mg/kg for 5 day	Counted by flow cytometry	ED50 values of 0.14, 0.08 mg/kg reducing cyst burden by 76–88%	Effective	Atovaquone	Doggett et al., 2012
						5 or 25 mg/kg for 16 day					
18	Spiramycin coadministered with metronidazole	Male BALB/c	ME49	Chronic	1000 tachyzoites orally	400 mg/kg daily for 7 days	Brain cysts counted	Metronidazole increased spiramycin brain penetration, causing a significant reduction of *T. gondii* brain cysts	Metronidazole alone showed no effect	–	Chew et al., 2012
						500 mg/kg daily					
19	New naphthoquinones, an alkaloid	Female Swiss-Webster	EGS	Chronic	10 tissue cysts orally	50 μg/mL of QUI-11, 100 μg/mL of either QUI-6 or QUI-11	Presence of tachyzoites in the peritoneal cavities and survival rates	The survival rates increased	Effective	Atovaquone	Ferreira et al., 2012
20	Prednisolone	Swiss albino	RH ME49	Acute, chronic	1 × 10^4^ tachyzoites, iP	235, 470, 705 mg/kg	Number of tachyzoites present	Greatly improved the number of tachyzoite, cyst forms in mice	No effective	–	Puvanesuaran et al., 2012
21	Salicylic acids compounds 14a, 14b	Swiss Webster	RH, RH-YFP, ME49	Acute	Oocysts orall gavage	100 or 25 mg/kg orally	Survival rates	Increased survival by 1 day	Effective	Pyrimethamine, sulfadiazine	Fomovska et al., 2012
22	Atorvastatin	Female Swiss Webster, BALB/c	RH TATi	Acute	5–20 tachyzoites i.p 10,000–100,000 tachyzoites	20 mg/kg/day ip	Plaque assays and containing tachyzoites in peritoneal fluid	Atorvastatin protect mice against death, cures a lethal infection	Effective	–	Li et al., 2013
23	Fusidic acid	Female BALB/c	Prugniaud	Acute	5 × 10^3^ or 5 × 10^4^ tachyzoites i.p	20 mg/kg	Parasite burdens, analyses of host cytokine, and survival rates	There was no statistically significant difference between mice treated with fusidic acid versus saline	No effective	Trimethoprim, sulfadiazine	Payne et al., 2013
24	FLZ combined with sulfadiazine, and pyrimethamine	CF1	RH	Acute	10^3^ tachyzoites	10 mg/kg/day of fluconazole with 40/1 mg/kg/day sulfadiazine, pyrimethamine	Survival rates	93% survival	Effective	Sulfadiazine, pyrimethamine	Martins-Duarte et al., 2013
25	Two naphthalene-sulfonyl-indole compounds	BALB/c	RH	Acute	2 × 10^6^ tachyzoites	25–800 μmol i.p	Survival rates, liver touch smears with giemsa stained	Both of the compounds was preserved	Effective		Asgari et al., 2013
26	Toltrazuril	lambs	ME49	Chronic	1 × 10^5^oocysts	20, 40 mg/kg orally 2 times, once every week	Presence of tissue cysts by histopathology, immunohistochemistry, and nested-PCR	Cyst presence was determined as 44.4%	Effective		Kul et al., 2013
27	Auranofin	Chicken embryos	RH	Acute	1 × 10^4^ tachyzoites chorioallantoic vein	1 mg/kg	Histopathology, immunohistochemistry, and qPCR	Significantly reduced parasite load	Effective	Pyrimethamine, sulfadiazine	Andrade et al., 2014
28	Spiroindolone	Mice	RH	Acute	2000 tachyzoites	100 mg/kg/day	Parasite burdens, measuring the fluorescence intensity	Reduced the parasite burden in mice by 90%	Effective	–	Zhou et al., 2014
29	6-Trifluoromethyl-2-thiouracil KH-0562^i^ and ATT-5126^j^	ICR female	RH	Acute	1 × 10^5^ tachyzoites	100 mg/kg KH-0562^i^ or ATT-5126^j^ orally	Measuring amount of the tachyzoites in mice ascites,LPO^k^, GSH^l^, ALT^m^, AST^n^ in mouse liver	LPO level-KH-0562 and ATT-5126 = 87.4 and 105.2 nmol/g	KH-0562 more effective	Pyrimethamine	Choi et al., 2014
30	Pyrazolopyrimidine-1294	BALB/c	RH Pru	Acute, chronic	10^5^ tachyzoites	100, 30 mg/kg/day for 5 days	Survival rates and number of *T. gondii* per ml	Decreasing the numbers of *T. gondii* tachyzoites at both 100, 30 mg/kg	Effective	–	Doggett et al., 2014
31	6-Trifluoromethyl-2-thiouracil KH-0562^i^, ATT-5126^j^	Female ICR	RH	Acute	1 × 10^5^ tachyzoites	100 mg/kg	Proteomic profiles of *T. gondii* tachyzoites	Decreased the amount of tachyzoites, mean numbers of tachyzoites = (66.8 ± 0.8) × 10^6^	Effective	Pyrimethamine	Choi et al., 2014
32	Cromolyn sodium, ketotifen	Balb/c	RH	Acute	4 × 10^5^ tachyzoites	Ketotifen 1, 2 mg/kg, cromolyn sodium 5, 10 mg/kg, ip	Inhibition evaluated under a light microscope with giemsa staining	After 60 min ketotifen at 2 mg/kg (69.83 ± 2.25 %), cromolyn sodium, at 10 mg/kg in (80.47 ± 2/49 %) had the best effect	Effective	–	Rezaei et al., 2014
33	Diclazuril plus atovaquone	CD1 mice	PTG Strain	Chronic	600 tachyzoites-i.p	65, 120 mg/kg diclazuril	Hematoxylin eosin,Giemsa,immuno histochemical staining	Combination diclazuril plus atovaquone was safe	Effective	–	Oz, 2014b
34	Diclazuril plus atovaquone	CD1 mice	PTG strain	Chronic	300, or 600 tachyzoites i.p	65, 120 mg/kg diclazuril	Hematoxylin and eosin, slides evaluated of colonic tissues	Combined therapy synergistically normalized pathology and to a lesser degree monotherapy	Effective	–	Oz, 2014a
35	Am80	BALB/c mice	RH, PLK	Acute	1 × 10 ^3^ tachyzoites i.p	1 mg/kg	Survival rates	Percent survival of mice increased statistically	Effective	–	Ihara and Nishikawa, 2014
36	Chitosan and silver nanoparticles	Swiss albino	RH	Acute	3.5 × 10^3^ tachyzoites i.P	100, 200 μg/ml	Parasite density and ultrastructural parasite changes	Statistically significant decrease in the mean number of the parasite count in the liver and the spleen	Effective	Pyrimethamine	Gaafar et al., 2014
37	Pyrimethamine/sulfadiazine	Female C57BL/6 mice	ME49	Chronic	20 cysts i.p	Pyrimethamine, sulfadiazine 4, 100 mg/kg daily for one month	Histology, qPCR, measured KP metabolites	Significant increases in these kynurenine pathway metabolites were observed in the brain at 28 days post-infection	Effective	–	Notarangelo et al., 2014
38	Pyrimethamine-loaded lipid-core nanocapsules	Female CF1 mice	RH	Acute	10^3^ tachyzoites	5.0–10 mg/kg/day	Surviving mice, cyst brain evaluation, bioassay urea, AST and ALP^o^	Survival rate higher than the animals treated with the same doses of non-encapsulated pyrimethamine	Effective	–	Pissinate et al., 2014
39	Atovaquone and astragalus combination	BALB/c	RH	Acute	2 × 10^4^/ml trophozoites	Atovaquone, astragalus 100, 0.075 mg/kg/day oral gavage	Peritoneal trophozoite numbers, IL-2, IL-12, IFN-γ^p^ levels were determined by ELISA	The number of trophozoites in the combination groups were found significantly lower than the number of trophozoites in the control group	Effective	–	Sönmez et al., 2014
40	Rolipram	Female Swiss albino mice	KSU strain	Chronic	20 tissue cysts	10 mg/kg daily for three weeks	Life expectancy, serum Alt, histopathology of liver and brain	Rolipram exerts a significant lowering effect on ALT levels, pathology	Partially effective	–	Afifi et al., 2014
41	Rolipram	Female Swiss albino mice	Low pathogenic strain	Chronic	20 tissue cysts	–	Tissue injury scoring, brain cyst count, specific Ig G titers, TNF- α^q^, IFN- γ and IL-12 assays	Significant reduction of TNFα (84.6%), IFN- γ (76.7%), IL-12 (71%)	Partially effective	–	Afifi and Al-Rabia, 2015
42	Triclosan (TS) and triclosan-loaded liposomal nanoparticles	Swiss strain Albino mice	RH HXGPRT (-)	Acute	10^4^ tachyzoites	150 mg/kg TS or 100 mg/kg TS liposomes	Mice mortality, peritoneal, liver parasite burdens	Reduction in mice mortality, parasite burden	Effective	–	El-Zawawy et al., 2015b
43	Sulfamethoxazole-trimethoprim (ST) associated with resveratrol	Male Swiss albino mice	VEG strain	Chronic	50 cysts containing bradyzoites	ST (groups B, F), free resveratrol (groups C,G) 0.5, 100 mg kg^−1^	Cyst counts in the brain, and histopathology analyses	Combination was able to reduce the number of cysts in the brain, inflammatory infiltrates in the liver, prevented the occurrence of hepatocytes lesions	Effective	Sulfamethoxazole, trimethoprim	Bottari et al., 2015
44	Ciprofloxacin derivatives (compounds 2, 4,5)	Female Swiss mice	RH	Acute	5 × 10^3^ tachyzoites i.p	25, 50, 100, or 200 mg/kg/day a single oral dose	Survival rate, determine the serum levels of urea and creatinine kinase	Increased mouse survival significantly, with 13–25% of mice surviving for up to 60 days post infection	Effective	–	Martins-Duarte et al., 2015
45	Triclosan (TS), TS liposomal	Swiss albino mice	ME49	Chronic	10 cysts	200, 120 mg/kg	Mortality,brain parasite burden	TS significant diminution in the parasite burden, great reduction in the infectivity power of *T.gondii* cysts	Effective	–	El-Zawawy et al., 2015a
46	2-(Naphthalene-2-γlthiol)-1H Indole 2-(naphhalene-2-ylthio)-1H-indole	BALB/c	RH	Acute	2 × 10^6^ tachyzoites exposed to the concentrations of the compound i.p.	25–800 μM for 1.5 h	Surviving mice, stained by PI and analyzed by fluorescence-activated cell sorting (FACS)	The longevity of mice was dose dependent. Five mice out of group 400 μmol and 3 out of group 800 μmol showed immunization to the parasite	Effective	–	Asgari et al., 2015
47	Propranolol	BALB/c	RH	Acute	1 × 10^3^ tachyzoites i.p	2, 3 mg/kg/day	Parasite load determined	In the pre-treatment group, propranolol combined with pyrimethamine was more effective	Effective	Pyrimethamine	Montazeri et al., 2015
48	Aripiprazole	BALB/c	Tehran strain	Chronic	50 tissue cysts, i.p	10, 20 mg/kg	Cysts counted in smears prepared from brain homogenate by optical microscope	No significant difference between mean logarithms of brain cyst numbers of aripiprazole groups compared with control	No effective	–	Saraei et al., 2015
49	Pyrimethamine (PYR) and sulphadiazine (SDZ) combined with levamisole and echinacea	BALB/c	RH	30 days after treatment	10^5^ tachyzoite i.p	PYR; 6.25, 12.5 SDZ; 100, 200 PYR, SDZ, levamisole; 2.5, echinacea; 130, 260 mg/kg/day oral treatment 24 h later for 10 days	Survival rates	Survival rate PYR+SDZ, and levamisole = 33.3% to 88.9%	Effective	Pyrimethamine, sulfadiazine	Köksal et al., 2016
50	Miltefosine	Swiss albino mice	RH ME49	Acute, chronic	2500 tachyzoites i.p 10 cysts orally	20 mg/kg for 5 days	Survival rates, tachyzoites count in the liver, spleen, cyst count and size in the brain ultra structural study, and histopathological study	Survival rate in acute = 30% Survival rate in chronic = 5%	No effective in acute. Partially effective in chronic	Sulphadizine	Eissa et al., 2015
						20 mg/kg/day					
						60 days post infection for 15 days					
51	Tetraoxanes	Female Swiss Webster	RH	Acute	10^2^ and 10^6^tachyzoite i.p	10 mg/kg/day, subcutaneously for 8 days	Survival rates and pathohistological analysis	Survival rate = 20 %	Effective	–	Opsenica et al., 2015
52	Guanabenz	BALB/c	ME49Prugniaud	Acute, chronic	10^4^ ME_49_or 10^6^ Pru tachyzoites, i.p	5 or 10 mg/kg repeated every 2 days	Survival of mice, qPCR	Enhanced survival, reduces cyst burdens in chronically infected mice	Effective	–	Benmerzouga et al., 2015
53	Fluphenazine and Thioridazine	BALB/c	Tehran strain	chronic	20 tissue cysts i.p	Thioridazine 10, 20, fluphenazine 0.06 mg/kg/ three days after inoculation for 3 weeks	The number of brain cysts	Drugs reduced the percent of cysts at higher dose compared to lower doses	Effective, not significant	Pyrimethamine	Saraei et al., 2016
54	Nitrofurantoin	Female ICR mice	RH	Acute	1 × 10^5^ tachyzoites	20, 50, and 100 mg/kg, orally once/day for 4 days	The numbers of tachyzoites in the peritoneal cavity, Hematology and biochemical parameters	The inhibition rate = 44.7% hematology indicators and biochemical parameters reduced by nitrofurantoin significantly	Effective	Pyrimethamine	Yeo et al., 2016
55	Dextran sulfate	Pigs	RH	Acute	1 × 10^6^ tachyzoites, intravenously	50–500 μg per head	host clinical, pathological, and immunological analyses	High-dose caused reversible hepatocellular degeneration of the liver	Effective	.	Kato et al., 2016
56	Propranolol	BALB/c	RH	Acute, chronic	1 × 10^3^ tachyzoites i.p	2, 3 mg/kg/day	Parasite load determined by qPCR, and survival rate	Decreased the parasite load in brain, eye, and spleen tissues	Effective	Pyrimethamine	Montazeri et al., 2016
57	Resveratrol and sulfamethoxazole-trimetropim	Male Swiss Webster	VEG	Chronic	50 cysts orally	Oral doses of 0.5 and 100 mg/kg/day	Counting brain cysts, tissue oxidant and antioxidant levels, and histopathology	A reduction on the number of cysts in the brain was observed	Co-administration more effective	Sulfamethoxazole-trimethoprim	Bottari et al., 2016
58	Compound 22 of sulfur-containing linear bisphosphonates	Webster mice	RH	Acute	20 or 100 or 5000 tachyzoites i.p	0.05, 0.1, 0.5, and 1 mg/kg of 22/ i.p. for 10 days	Survival rate	ED_50_= 0.02 mg/kg	Effective	.	Szajnman et al., 2016
59	Compound32 (TgCDPK1 inhibitor)	Female CF-1 CBA/J	RH ME49	Acute, chronic	less than 100 tachyzoites/mL	20 mg/kg for	The numbers of tachyzoites in spleen, brain/ and the number of brain cysts	Reducing infection in spleen and brain (99%, 95%) 88.7% reduction of brain cyst	Effective	.	Vidadala et al., 2016
						5 days/ oral gavage					
						30 mg/kg for 14 days					

a *2-hydroxy-3-(1_-propen-3-phenyl)-1,4-naphthoquinone*.

b *Indirect immunofluorescence antibody test*.

c *Reverse transcription polymerase chain reaction*.

d *Polymerase chain reaction*.

e *Quantitative Polymerase chain reaction*.

f *Fluconazole*.

g *Itraconazole*.

h *1-Hydroxy-2-Alkyl-4(1H) Quinolone*.

i *6-trifluoromethyl-2-thiouracil*.

j *3-[{2-((E)-furan-2-ylmethylene) hydrazinyl} methylene]-1, 3-dihydroindol-2-one*.

k *Lipid peroxidation*.

l *Glutathione-S-transferase*.

m *Alanine aminotransferase*.

n *Aspartate amino transferase*.

o *Alkaline phosphatase*.

p *Interferon gamma*.

q *Tumor necrosis factor*.

### Cell culture

The cell cultures used in *in vitro* studies were mostly human foreskin fibroblast (HFF; 39 studies), LLCMK2 (12 studies), Vero (11 studies), Hela (6 studies), mouse macrophage cell line (J774A.1) (5 studies), and MRC-5 (2 studies; Table 3).

### Laboratory animals

*T. gondii* can infect most warm-blooded animals, and is studied in different animal models depending on the nature of the investigation (Szabo and Finney, 2016). The animal model used in studies was mostly mice (16 studies BALB/c and 19 studies Swiss-Webster). In murine models of acute toxoplasmosis, some medicines were protective even when administered at low dosages. But some drugs despite of their excellent *in vitro* activity were poorly protective in murine models with acute toxoplasmosis (Payne et al., 2013).

### Diagnostic tests and evaluation methods

The present review outlines the results of *in vitro* screening methods including morphological assay, incorporation of [3H] uracil assay, plaque assays, enzyme-linked immunosorbent assay (ELISA), colorimetric micro titer assay (b-galactosidase assay), flow cytometric quantification assay, and cell viability assay. Numerous versions of fluorescent proteins have been expressed in *T. gondii* (Kim et al., 2001). The reporter genes used *in vitro* and *in vivo* studies were the green fluorescent protein (GFP) and yellow fluorescent protein (YFP). Parasites expressing fluorescent proteins can also be analyzed and sorted by flow cytometry. This technology used for drugs screening in 10 studies.

Details about the diagnostic methods and drug dosage under *in vivo* conditions are shown in Table 4. Also, a comprehensive list of drugs/compounds evaluated against *T. gondii* with regard to IC_50_ is illustrated in Table 5.

**Table 5:** **A comprehensive list of drugs/compounds evaluated against ***T. gondii*** with regard to IC_**50**_**.

**Drug**	**IC**_**50**_ **(**μ**M)**			**References**
	**<1**	**1–5**	**5–10**	
Novel quinuclidine	+			Martins-Duarte et al., 2006
Novel ferrocenic atovaquone derivatives	Atovaquone (PLK strain)	2d, 2e, 2f		Baramee et al., 2006
SAHA^a^, SBHA^b^, Scriptaid, Trichostatin A	Scriptaid			Strobl et al., 2007
	Trichostatin A			
	SAHA			
	SBHA			
Pyridinylimidazoles SB203580 and SB202190	RWJ67657, (ME49 strain)	SB202190	SB203580	Wei et al., 2007
		SB203580		
		RWJ68198, (ME49 strain)	RWJ68198, (RH strain)	
		RWJ67657, (RH strain)		
1-hydroxy-2-dodecyl-4(1H) quinolone	+			Saleh et al., 2007
Fluorine-containing aryloxyethyl thiocyanate derivatives		Compound 1, 3, 9	Compound 10	Liñares et al., 2007
Novel diamidine analog	+			Leepin et al., 2008
Pyrimethamine, sulfadiazine, atovaquone	+			Meneceur et al., 2008
Novel triazine JPC-2067-B	+			Mui et al., 2008
2-alkylaminoethyl-1,1-bisphosphonic acids		Compound 19	Compound 14, 17	Szajnman et al., 2008
Itraconazole	+			Martins-Duarte Edos et al., 2008
Thiolactomycin analog		Compound 5, 6	Compound 2	Martins-Duarte et al., 2009
Fluconazole (FLZ)		FLZ (48 h)	FLZ (24 h)	Martins-Duarte et al., 2010
1-Hydroxy-2-Alkyl-4(1H) Quinolone derivatives	+			Bajohr et al., 2010
Haloperidol, clozapine, fluphenazine, trifluoperazine, thioridazine		+		Goodwin et al., 2011
Novel azasterols	Compound 3 (48 h)	Compound 1 (48 h), 2, 3 (24 h)	Compound 1 (24 h)	Martins-Duarte et al., 2011
Endochin-like quinolones	+			Doggett et al., 2012
Pterocarpanquinone		+		Portes Jde et al., 2012
New naphthoquinones (QUI), an alkaloid	QUI-11			Ferreira et al., 2012
	Liriodenine			
Di-cationic, pentamidine-analog	+			Kropf et al., 2012
Fuconazole combined with sulfadiazine and pyrimethamine	Pyrimethamine		+	Martins-Duarte et al., 2013
Antipsychotic drugs and valproate		Fluphenazine	Zuclopenthixol	Fond et al., 2014
		Thioridazine		
Fusidic acid			+	Payne et al., 2013
Ivermectin and sulphadiazine	Ivermectin		Sulphadiazine	Bilgin et al., 2013
Novel ruthenium complexes,(compounds 16 and 18)	+			Barna et al., 2013
Auranofin	+			Andrade et al., 2014
6-Trifluoromethyl-2-thiouracil		+		Choi et al., 2014
200 drug-like, 200 probe-like compounds of Malaria Box	MMV007791	MMV007881		Boyom et al., 2014
		MMV007363		
		MMV006704		
		MMV666095		
		MMV020548		
		MMV085203		
Quinoline derivatives	8-Hydroxyquinoline, A 11, A14, A18, B11, B12, B15, B23, B24	A2-6, A12, A15—17, A23, B16, B22, B26, B27, B29, Chloroquine	Quinoline	Kadri et al., 2014
			2-chloroquinoline	
			5-Nitroqu	
			Inoline Quinoline	
			N-oxide hydrate A7, B18	
Bumped Kinase Inhibitor 1294	+			Doggett et al., 2014
Salicylanilides	3i, 3j, 7a, 14a, 14b			Fomovska et al., 2012
Antiretroviral compounds		Atazanavir	Fosamprenavir	Monzote et al., 2013
		Ritonavir	Nelfinavir	
		Saquinavir		
Spiroindolone		+		Zhou et al., 2014
Ciprofloxacin derivatives	Compound 2, 5	Compound 4		Dubar et al., 2011
Thiazolidin-4-one derivatives	12A	27, 34 A	36 A	D'Ascenzio et al., 2014
N6-benzyladenosine analog			Compound 11 e, g, j, n, o, q, u, v	Kim et al., 2007
Naphthoquinone derivative	LQB151 (48 h)	LQB94		da Silva et al., 2015
		LQB151 (24 h)		
		LQB150 (24, 48 h)		
Oryzalin analogs	Compound 6a, h, i, 14a, 18a, b, c	Compound 6b, g, j, I, n, 12	Compound 6m, 14b	Endeshaw et al., 2010
94 compounds		+		Dittmar et al., 2016
6-(1,2,6,7-tetraoxaspiro[7.11] nonadec-4-yl)hexan-1-ol (N-251)		+		Xin et al., 2016

a *Suberoylanilide hydroxamic acid*.

b *Suberic bishydroxamic acid*.

## Discussion

The aim of this systematic review was to investigate the *in vitro* and *in vivo* effects of anti-*Toxoplasma* drugs and synthetic compounds, from 2006 to 2016. The current anti-*T. gondii* chemotherapy is deficient; as it is not well-tolerated by immunocompromised patients and cannot completely eradicate tissue cysts produced by the parasite (Rodriguez and Szajnman, 2012). Therefore, developing new, safe, effective, and well-tolerated drugs with novel mechanisms of action could be a global priority (Lai et al., 2012). An ideal drug for prophylaxis and/or treatment of toxoplasmosis would show effective penetration and concentration in the placenta, transplacental passage, parasiticidal properties vs. the different parasitic stages, penetration into cysts, and distribution in the main sites. No available drug fulfills these criteria (Derouin et al., 2000; Montoya and Liesenfeld, 2004).

Thus, the findings of the present systematic review article encourage and support more accurate investigations for future to select new anti-*Toxoplasma* drugs and strategies in designing new targets with specific activity against the parasite.

### Activities of anti-*toxoplasma* clinically available drugs

With growing parasite resistance to therapeutic drugs and in the absence of a vaccine, to increase the effectiveness of drugs, various changes have been made in construction of the clinically available medicines. Thus, the activity of new formulations of clinically available drugs against *T. gondii* should be evaluated to find alternative treatments for toxoplasmosis (da Cunha et al., 2010).

Interestingly, encapsulation of pyrimethamine improved the efficacy and tolerability of this drug against acute toxoplasmosis in mice and can be considered as an alternative for reducing the dose and side effects of pyrimethamine (Pissinate et al., 2014). Recently, researchers reported that computational analysis of biochemical differences between human and *T. gondii* dihydrofolate reductase enabled the design of inhibitors with both improved potency and selectivity against *T. gondii* (Welsch et al., 2016). El-Zawawy et al. reported that incorporating triclosan into in the lipid bilayer of liposomes allowed its use in lower doses, which in turn, reduced its biochemical adverse effects (El-Zawawy et al., 2015b). In another study, sodium dodecyl sulfate (SDS)-coated atovaquone nanosuspensions (ANSs) considerably increased the therapeutic efficacy against experimentally reactivated and acquired toxoplasmosis by improving passage of gastrointestinal and blood-brain barriers. Accordingly, coating of ANSs with SDS may improve the treatment of toxoplasmic encephalitis and other cerebral diseases (Shubar et al., 2011).

Also, various studies showed that a number of drugs were investigated for the mechanisms of action summarized in Table 2 and Figure 2. One study discussing the metabolic differences between the host and the parasite noted that dihydrofolate reductase, isoprenoid pathway, and *T. gondii* histone deacetylase are promising molecular targets (Rodriguez and Szajnman, 2012).

Novel triazine JPC-2067-B (4, 6-diamino-1, 2-dihydro-2, 2-dimethyl-1-(3′(2-chloro-, 4-trifluoromethoxyphenoxy)propyloxy)-1, 3, 5-triazine), the anti-folate medicines, is highly effective against *T. gondii* with an IC_50_ of 0.02 μM, which is more efficacious than pyrimethamine and has *in vitro* cidal activity. Additionally, pro-drug JPC-2056 (1-(3′-(2-chloro-4-trifluoromethoxyphenyloxy) propyl oxy)-5-isopropylbiguanide) is effective *in vivo* when administered orally (Mui et al., 2008). Moreover, histone deacetylase is potentially a very important drug target in *T. gondii*, since scriptaid and trichostatin A had the highest effect against *T. gondii* tachyzoite proliferation with the IC_50_ of 0.039 and 0.041 μM, respectively (Strobl et al., 2007). For promising anti- *T. gondii* drugs/compounds, assessment of their ability to control parasite growth is a key step in drug development (McFarland et al., 2016).

A large number of research papers suggested that the apicoplast represents a potential drug target for new chemotherapy, as it is essential to the parasite and it is absent in host cells. Functions of the apicoplast include fatty acid synthesis, protein synthesis, DNA replication, electron transport, and heme biosynthesis (Yung and Lang-Unnasch, 2004). Some of the drugs evaluated against *T. gondii* are shown to act in the apicoplast such as thiolactomycin, triclosan (TS), azithromycin, fusidic acid, ciprofloxacin, and quinoline derivatives (Costa et al., 2009; Martins-Duarte et al., 2009, 2015; Payne et al., 2013; Kadri et al., 2014; El-Zawawy et al., 2015b).

In *T. gondii*, FAS-II enzymes are present in the apicoplast and are essential for its survival. The key enzyme in this process is the ENR enzyme, which is not found in mammals (Surolia and Surolia, 2001). This enzyme catalyzes the last reductive step of the type II FAS pathway. The TS, which inhibits type II FAS, significantly reduced mice mortality, parasite burden, as well as viability and infectivity of tachyzoites and cysts harvested from infected treated mice and their brains. Accordingly, TS is proved as an effective, promising, and safe preventive drug against acute and chronic murine toxoplasmosis. Liposomal formulation of TS enhanced its efficacy and allowed its use at a lower dose (Surolia and Surolia, 2001; El-Zawawy et al., 2015a,b). Among apicoplast pathways, DNA replication is an important potential chemotherapeutic target. Fluoroquinolones are the known DNA replication inhibitors that target prokaryotic type II topoisomerases (Collin et al., 2011). In two studies, researchers showed that derivatives of the antibiotic ciprofloxacin, a fluoroquinolone, are active against *T. gondii* tachyzoites both *in vitro* and *in vivo* (Neville et al., 2015). While all mice treated with ciprofloxacin died by day 10 post-infection, some mice treated with ciprofloxacin derivatives remained alive for at least 60 days, suggesting that ciprofloxacin derivatives cured *T. gondii* infection in treated mice (Dubar et al., 2011; Martins-Duarte et al., 2015).

### Anti-*toxoplasma* activities of new synthetic compounds

There are numerous reports on efficacy of many new synthetic compounds with a focus on identifying drug candidates with innovative and acceptable profiles against *T. gondii*. The anti-coccidial effect of 1-[4-(4-nitrophenoxy) phenyl] propane-1-one (NPPP), a synthetic compound, was studied both *in vitro* and *in vivo*. Treatment with NPPP showed anti-*Toxoplasma* activity *in vitro* with a lower EC_50_ value than pyrimethamine. In ICR mice infected with *T. gondii*, oral administration of NPPP for 4 days showed statistically significant anti-*Toxoplasma* activity with lower number of tachyzoites than those of the negative control (Choi et al., 2015).

In a study by Kadri et al. anti-*Toxoplasma* properties of 58 newly synthesized quinoline compounds were evaluated. A significant improvement in anti-*Toxoplasma* effect among quinoline derivatives was detected in B11, B12, B23, and B24. Among these compounds, B23 was the most effective compound with the IC_50_ value of < 1 μM, displaying its anti-*Toxoplasma* effects and ability to cause the disappearance of the apicoplast (40–45% of the parasites lost their apicoplasts; Kadri et al., 2014).

In a study by Boyom et al. the strategy adopted was to repurpose the open access Malaria Box to identify chemical series active against *T. gondii*. The results showed that the most interesting compound was MMV007791, a piperazine acetamide, which has an IC_50_ of 0.19 μM. This compound is novel for its anti-*Toxoplasma* activity, and of course, further studies on the rates and mechanisms of compound action will elucidate these considerations (Boyom et al., 2014).

Tetraoxanes, anti-cancer molecules, were tested *in vivo* against *T. gondii*. Subcutaneous, administration of a 10 mg/kg/day dose of derivative 21, for 8 days allowed the survival of 20% of infected mice, demonstrating the high potential of tetraoxanes for the treatment of *T. gondii* (Opsenica et al., 2015).

In another study by Moine et al. researchers evaluated *in vitro* anti-*T. gondii* activity of 51 compounds with a biphenylimidazoazine scaffold. Eight of these compounds displayed highly potent activity against *T. gondii* growth *in vitro*, with 50% effective concentration (EC_50_) below 1 mM, without demonstrating cytotoxic effects on human fibroblastic cell at equivalent concentrations. However, these compounds have to be evaluated in animal models so as to confirm their *in vivo* activity (Moine et al., 2015a).

Several pathways were characterized and shown to differ significantly from those of the mammalian host cells, thus, revealing an attractive area for therapeutic intervention. 1-Hydroxy-2-Alkyl-4 (1H) quinolone derivatives inhibit the fourth step of the essential *de novo* synthesis of pyrimidine, which uses ubiquinol reduction as an electron sink for dihydroorotate oxidation (Saleh et al., 2007). Also, newly synthesized bisphosphonates interfere with the mevanolate pathway, which leads to the synthesis of sterols and polyisoprenoid compounds that are important for parasite survival (Shubar et al., 2008).

Interestingly, Kamau et al. identified novel kinases that are integral to essential pathways, elucidating their mechanism of action and ultimately, identifying new drug targets (Kamau et al., 2012). In that study, 527 compounds were evaluated *in vitro;* also, they assessed the impact of the inhibitory compounds C1, C2, C3, and C5 in mouse models of toxoplasmosis. C2 was found quite effective in decreasing the parasite burden and increasing mice survival. These results should be considered with caution, since there are a number of factors are at play in whether a compound will be *in vivo* effective, such as solubility *in vivo*, access to different tissues, and host metabolic processes (Kamau et al., 2012). In a recent study, Dittmar et al. screened a collection of 1,120 compounds, 94 of which were blocked parasite replications with IC_50_ of <5 μM. These data suggest that tamoxifen restricts *Toxoplasma* growth by inducing xenophagy or autophagic destruction of this parasite (Dittmar et al., 2016). According to a new study, *in silico* screening is useful, particularly in the identification of molecular targets in the laboratory. Fernandez et al. synthesized VAM2 compounds (7-nitroquinoxalin-2-ones), based on the design obtained from an *in silico* prediction with the software TOMOCOMD-CARDD. From the group of VAM2 compounds, Fernandez et al. chose VAM2-2 with an IC_50_ of 3.3 μM against *T. gondii*. However, more studies are required to evaluate its effect on the cysts formed by of the parasite and in animal models of toxoplasmosis (Fernández et al., 2016).

### Activity of drugs, compounds, and combined therapy against cysts

An ideal drug against toxoplasmosis should not only be effective against the proliferative stage of the parasite but also exert dual activity against the tissue cyst stage and penetration into cysts (Benmerzouga et al., 2015). Currently, there is no approved therapy that eliminates the tissue cysts responsible for chronic infection (Innes, 2010). Derouin reported that among the drugs commonly used in humans, only atovaquone and azithromycin were found effective after long-term incubation. Besides, arpinocid-N-oxyde, an anticoccidial for veterinary use, was efficient at a high dosage (Derouin, 2005).

Recently, investigators have focused on guanabenz for *in vivo* studies, as guanabenz inhibitor of eIF2a dephosphorylation, is already an food and drug administration (FDA) approved drug and has excellent solubility with good penetration into the CNS. The results of that study show that guanabenz (5 mg/kg/day) not only protects mice against acute toxoplasmosis, but also reduces 69% of the number of brain cysts in chronically infected animals. This finding suggested that guanabenz can be repurposed into an effective antiparasitic with a unique ability to diminish tissue cysts in the brain (Benmerzouga et al., 2015).

Another study showed that miltefosine had no efficacy in controlling acute toxoplasmosis after 5 days of treatment; however, a 15-day treatment against the established chronic stage led to a 78% reduction of cysts in the brain. Additionally, the remaining cysts were noticeably smaller upon microscopic examination, suggesting that the drug effectively penetrates the blood-brain barrier, and that extension of treatment time may produce greater effects (Eissa et al., 2015).

In another study by Maubon et al. FR235222 and its derivatives were identified as new lead compounds for use against acute and chronic toxoplasmosis both *in vitro* and *in vivo*. *In vivo* experiments indicated that FR235222, as a histone deacetylase inhibitor, is able to access the bradyzoites within the cyst. The ability of FR235222 to permeate the membrane wall is a major advantage for crossing the blood-brain barrier and CNS tissue, where *Toxoplasma* cysts are located. This opens a promising way to develop drugs that are selective against *Toxoplasma* and those that have sterilizing activity, especially in patients with cysts, who are at risk for reactivating acute toxoplasmosis (patients with HIV infection, hematological malignancies, or transplantation). Still, effectiveness of FR235222 against chronically infected mice remains to be directly demonstrated *in vivo* (Maubon et al., 2010).

In a new study Vidadala et al. identified compounds 32 (*T. gondii* calcium-dependent protein kinase 1 inhibitor) a promising lead for the development of a new antitoxoplasmosis therapy. Compounds 32 is CNS-penetrant and highly effective in acute and latent mouse models of *T. gondii* infection, significantly reducing brain cysts by 88.7% (Vidadala et al., 2016).

Many studies reported anti- *Toxoplasma* effects of different drugs combination with novel compounds. The compound 2-hydroxy-3-(1′-propen-3-phenyl)-1, 4-naphthoquinone (PHNQ6), (50 mg/kg/day) combined with sulfadiazine showed reduction or elimination of brain cysts *in vivo* (Ferreira et al., 2006). In another study that coadministered spiramycin and metronidazole, spiramycin, did not reach effective concentrations in the brain due to the presence of the efflux transporters multidrug-resistant protein 2, and P-glycoprotein. Metronidazole increased brain penetration of spiramycin, causing a significant reduction of *T. gondii* brain cysts, with potential clinical translatability for chronic toxoplasmosis treatment. According to the reports, combination therapy leads to faster recovery, less relapse, lower doses of drugs, and fewer side effects of the disease. Furthermore, such combinations are highly promising to develop a drug that is able to eliminate the cyst stage of the parasite, and thus, efficiently impairs relapse of the disease (Chew et al., 2012; Martins-Duarte et al., 2013).

### Activity of drugs against congenital toxoplasmosis

In pregnant women, current toxoplasmosis treatment is based on the administration of spiramycin or a drug combination such as sulphadiazine-pyrimethamine-folinic acid (SPFA) in cases of confirmed fetal infection. However, these drugs are not well-tolerated and present many adverse effects due to their toxic effects to the host (Degerli et al., 2003).

Degerli et al. evaluated the effectiveness of azithromycin, artemisia annua infusion, spiramycin, and SPFA in Calomys callosus, such as model of congenital toxoplasmosis. The results demonstrated that the treatment of pregnant *C. callosus* with azithromycin was effective for inhibiting the vertical transmission of *T. gondii* ME49 strain, suggesting that it may be an alternative drug of choice for the treatment of congenital infection, since it is able to inhibit fetal infection and offers new perspectives for the treatment of congenital toxoplasmosis. Azithromycin is one of the new generation macrolides with numerous advantages. Mechanism of action of azithromycin is based on the inhibition of protein synthesis in both *T. gondii* tachyzoite and bradyzoite stages (Degerli et al., 2003), but it may present limited effectiveness against *T. gondii*, requiring high drug concentrations (Costa et al., 2009). In another study, Oz et al. reported that combined atovaquone and diclazuril therapy is a novel synergistic prophylactic and therapeutic approach to fetal maternal toxoplasmosis (Oz, 2014a). Atovaquone, an inhibitor of mitochondrial electron-transport processes, is an FDA-approved toxoplasmosis therapy but not for use in congenital toxoplasmosis treatment (Oz, 2014a). Another compound, diclazuril, and its related benzeneacetonitriles have long been used in the treatment and prevention of poultry and livestock coccidiosis. In addition, it is known to be a safe compound at therapeutic dose levels (Assis et al., 2010).

### Adverse effects of drugs

However, anti-*Toxoplasma* effects of drugs/compounds were reported in many trials, but prednisolone increased the number of tachyzoites and bradyzoites in immunosuppressed infected mice (Puvanesuaran et al., 2012). In addition, betamethasone can escalate the invasion of tachyzoites, in cell culture. It could be suggested that patients under prolonged use of betamethasone and prednisolone should be protected against *T. gondii* infection. Also, if individuals receiving betamethasone are infected with *T. gondii*, interferon-gamma may be used to reduce the invasion of tachyzoites (Ghaffarifar et al., 2006).

## Conclusions

As current chemotherapy against toxoplasmosis is still not satisfactory, the development of well-tolerated and safe specific immunoprophylaxis in relaxing the need of dependence on chemotherapeutics is a highly valuable goal for global disease control. Immunotherapeutics strategies for improving toxoplasmosis control could either be a vaccine which would induce strong protective immunity against toxoplasmosis, or passive immunization in cases of disease recrudescence. However, with the increasing number of high-risk individuals, such as immunocompromised patients, and absence of a proper vaccine, continued efforts are necessary for the development of novel treatment options against *T. gondii*. Some of the novel compounds reviewed here may represent good starting points for the discovery of effective new drugs. In further bioinformatic and *in silico* studies are needed in order to identify new potential toxoplasmicidal drugs.

## Author contributions

AD and MS conceived the idea for this review. MM and SS searched the databases for potentially eligible articles based on their titles and abstracts. AD and MM participated in the study design and wrote the manuscript. SM and EA critically reviewed the manuscript. All authors read and approved the final manuscript for publication.

### Conflict of interest statement

The authors declare that the research was conducted in the absence of any commercial or financial relationships that could be construed as a potential conflict of interest.
